# Helping Blind People Grasp: Evaluating a Tactile Bracelet for Remotely Guiding Grasping Movements

**DOI:** 10.3390/s24092949

**Published:** 2024-05-06

**Authors:** Piper Powell, Florian Pätzold, Milad Rouygari, Marcin Furtak, Silke M. Kärcher, Peter König

**Affiliations:** 1 Institute of Cognitive Science, University of Osnabrück, 49069 Osnabrück, Germanyfpaetzold@uos.de (F.P.); mrouygari@uos.de (M.R.); silke.kaercher@uos.de (S.M.K.); pkoenig@uos.de (P.K.); 2 FeelSpace GmbH, 49069 Osnabrück, Germany; 3 Department of Neurophysiology, University Medical Centre Hamburg-Eppendorf, 20251 Hamburg, Germany

**Keywords:** blindness, visual impairment, assistive technology, tactile bracelet, grasping, sensory augmentation

## Abstract

The problem of supporting visually impaired and blind people in meaningful interactions with objects is often neglected. To address this issue, we adapted a tactile belt for enhanced spatial navigation into a bracelet worn on the wrist that allows visually impaired people to grasp target objects. Participants’ performance in locating and grasping target items when guided using the bracelet, which provides direction commands via vibrotactile signals, was compared to their performance when receiving auditory instructions. While participants were faster with the auditory commands, they also performed well with the bracelet, encouraging future development of this system and similar systems.

## 1. Introduction

Simple grasping remains a challenge for many of the 338 million people worldwide living with severe visual impairment or blindness [1]. Most humans master grasping at an early age, as it is crucial to successful daily life and critical to a plethora of simple everyday actions, from moving objects to holding hands [2,3,4,5]. But, as vision is crucial to guiding the movements needed for grasping [6,7,8], those with a visual impairment struggle with this basic skill [9], leaving a significant segment of the population with a limited ability to participate fully in activities others take for granted [10]. Thus, it is critical to develop tools to assist this population in grasping and other activities vital to daily life.

Understanding why grasping can be uniquely challenging for the visually impaired begins with understanding the task. There are two competing models for how grasping is performed in humans—the visuomotor channels model [7,11] and the double-pointing model [12]. The former divides the movement into two parts—the reach, or the movement of the hand towards the target object, and the grasp, or the actual opening and closing of the hand in order to grip that object. In contrast, the double-pointing model combines these steps and views grasping as a smooth, continuous process consisting of the movement of the individual fingers toward target positions on the object. The debate between the two models is ongoing, with considerable evidence available for both accounts (for a review of the models, see [8]). However, the critical role of visual input in controlling the grasping movement is clear in both [7,8]. Although physically capable of conducting the movements involved in grasping, visually impaired individuals lack this crucial visual element, making grasping challenging [9,13].

Many sensory substitution devices have already been developed to assist the visually impaired with various activities by attempting to restore general-purpose vision (e.g., [14,15,16]). Such devices enable the sampling of sensory information through a modality other than the natural channel for that type of stimulus, for example, functionally replicating vision with auditory (e.g., [17]) or haptic (e.g., [18]) feedback. Several devices have achieved success in allowing their users to ”see” their surroundings by substituting feedback from other senses for the missing visual channel (e.g., [17,18,19,20]). However, while these and other sensory substitution devices allow the user to independently regain some functions normally handled through vision without the assistance of other people, they are not presently widely adopted or commercially available. One reason for this is that sensory substitution devices with auditory interaction have the disadvantage of obstructing the auditory channel, which serves as the primary source of information for visually impaired individuals [21]. Further, the broader family of sensory substitution devices requires extensive user training before being effective for users, and devices that seek to restore general vision provide a user with only a basic summary of what is in their environment, rather than the more precise information needed for more specific tasks (e.g., [15,16]). Thus, the development of more specialized tools tailored to help with more particular tasks is of great interest.

A group of such devices already exists (e.g., [22,23,24]) and uses the principle of sensory augmentation [25] to enhance, rather than replace, users’ abilities. In contrast to sensory substitution, sensory augmentation does not intend to restore one sense fully but, rather, to extend an existing sense or create a new sense to replace a missing or damaged one. The generation of new sensorimotor contingencies can realize such a process by introducing new rules governing functional relations between sensory stimulation and motor actions [26]. For example, the feelSpace (Osnabrück, Germany) naviBelt [27] orients users to cardinal north or along a predefined route by guiding them with 30 vibration motors attached to a belt around their waist, therefore offering learnable, non-auditory, and task-oriented assistance [28]. Unfortunately, such devices are still relatively scarce, with those intended to support grasping being even more rare. An ideal device specifically for grasping would, therefore, be one that maintains independent use, keeps the auditory channel unobstructed, needs minimal training, and tailors the information provided to precisely guide users’ movements toward the objects they wish to interact with.

The range of devices aimed at assisting grasping is still limited, but the number of devices with the goal of assisting at least one stage of the grasping movement is growing rapidly. For example, PalmSight [29] processes visual information from the camera placed on the user’s palm and provides tactile feedback using vibrating motors positioned on the back of the same hand. FingerSight [30] employs a similar method but with both motors and cameras placed on the index finger, and the tactile glove developed by de Paz et al. [31] signals an object’s shape and distance from the hand via the intensity of vibrations of motors put on the index finger and thumb. But, although these devices maintain the ability of the user to act independently from others and successfully assist with grasping objects, they have critical shortcomings that open avenues for improvement. Their placement on the hand, for a start, has the disadvantage of potentially interfering with the user’s ability to grasp some objects, and they also narrowly focus on the grasping movement itself, neglecting the guidance of the hand to the target object, which is, in fact, the aspect of grasping that is most challenging for the visually impaired. In the current work, therefore, we aimed to create a solution that leaves the auditory channel unobstructed while providing precise information to the user in order to guide their hand to objects before allowing them to use their intact sensorimotor ability to grasp the object after locating it.

Here, we present a novel device, a tactile bracelet adapted from the feelSpace naviBelt [27]. To leave the hand free, we located our device on the wrist, which still provides sufficient spatial resolution for users to differentiate between the four motors for the respective up, down, left, and right movement directions. The activation of the respective motors guides the user’s hand to the approximate location of the target object, and the participant is then allowed to use their natural sensorimotor abilities to complete the grasp, thus keeping the device focused on the navigation element of grasping that is most challenging for the visually impaired population. Such a device could help visually impaired and blind people gain a larger degree of independence in scenarios where they might be forced to rely more heavily on other people. One such scenario is searching for and grasping a product in an unfamiliar supermarket, where an additional person might need to locate the items that a visually impaired individual wishes to buy and place them in the shopping cart for them. To evaluate whether tactile commands could provide a foundation for increased autonomy in such scenarios, in this case by enabling a blind user to locate and obtain a desired item on a shelf on their own, but without being a slower navigational aid than the more familiar method of following information communicated auditorily, we first conducted a study with blindfolded participants to validate the viability of tactile signals sent via a bracelet to guide grasping movements. We first address this primary research aim by comparing the response time with the bracelet to the response time with auditory commands before subsequently validating our findings with the target group of blind people and finally exploring a fully closed-loop paradigm with a small qualitative pilot study and a preliminary AI control system to demonstrate independent use. In the following sections, we present our testing procedure and results, demonstrating the bracelet as a time-efficient and tractable replacement for auditorily guided hand orientation during grasping.

## 2. Materials and Methods

### 2.1. Participants

The device was tested with thirty normally sighted but blindfolded participants (gender: 17 female, 12 male, one diverse; age: M = 24.5 years, SD = 3.5 years; one left-handed) in the main study, two visually impaired participants in the validation pilot study with the target group, and a small group of sighted mock users in the AI control pilot study. Two participants had previous experience using the tactile bracelet from a prior thesis study [32], but the device was novel to most of the participant pool. The blindfolded participants were students in the Cognitive Science program at the University of Osnabrück at the time of participation. They received either program credit or monetary compensation of 12 euros per hour.

### 2.2. Technical Setup

**Figure 1:**
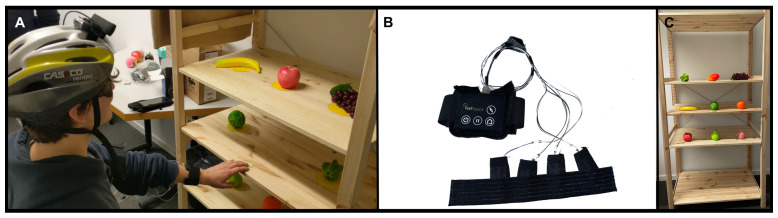
Participants wore the feelSpace tactile bracelet (**B**) along with a camera attached to a helmet (**A**), and they were guided to target objects on the shelf (**C**).

The feelSpace tactile bracelet (Figure 1B) is worn on the wrist of the dominant hand (the right hand for all but one of the participants in the current study), and it contains four vibration motors indicating each of the four directions (up, down, left, and right), respectively, with the motors providing vibrotactile input at $0^{\circ }$, $90^{\circ }$, $180^{\circ }$, and $270^{\circ }$ around the participant’s wrist. It is important to mention that the use of the device currently requires a fixed wrist orientation, as the tactile information does not adapt to hand orientation. The vibration motors of the bracelet are connected to a flexible strap, enabling adjustment to the participant’s wrist. They are controlled via a control box secured to the participant’s upper arm, which receives commands from the experimenter’s computer. To test the operation of the bracelet guided via a camera view of the scene (the ultimate paradigm that the bracelet will be used in), the experimenter operated the bracelet remotely from the other side of the experiment room, with their only view of the target objects coming from a helmet-mounted webcam worn by the participant. Participants were seated in front of a shelf on which nine target objects (artificial fruits) were arranged equidistantly (Figure 1A,C). By noting the positions of the participant’s hand and the target objects through the webcam feed and sending the appropriate commands to the tactile bracelet, the experimenter could guide the participant’s hand toward a target object on the shelf.

### 2.3. Experimental Procedure

**Figure 2:**
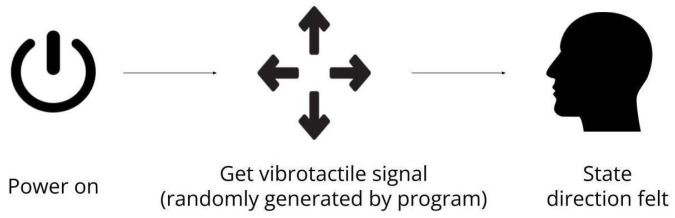
Conceptual representation of the localization task.

To familiarize the participants with the bracelet and evaluate their ability to differentiate between the vibrations of the four motors, we first conducted a localization task. For this task, participants sat at a table and rested their fingertips on the edge, ensuring that no part of the bracelet was touching the table. They then completed up to three blocks of 16 trials each, with a random motor vibrating on each trial. The participant was instructed to say which motor they thought had vibrated (Figure 2). Participants were required to achieve an accuracy of 90% before proceeding to the next stage of the experiment. They could repeat the localization task to achieve this level a maximum of three times. If a participant’s accuracy was below the cutoff, the bracelet was adjusted on their wrist to a position that better allowed them to distinguish between the motors. All participants achieved or surpassed the cutoff accuracy within two attempts at the task, with the majority achieving this in one attempt.

**Figure 3:**
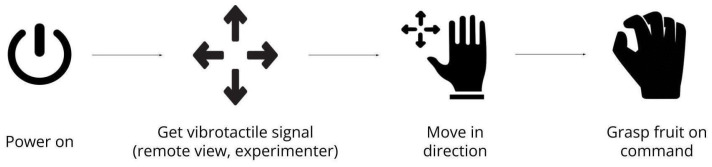
Conceptual representation of the grasping task.

Following the localization task, participants completed a grasping task to allow us to compare their grasping performance as guided via the tactile bracelet to their performance in a baseline condition in which we guided them with auditory commands. Participants put on a blindfold before completing 16 blocks of 9 grasping trials each, 8 blocks of which they completed with the bracelet and 8 with auditory commands, the first block being a practice block in both conditions. In the tactile condition, the experimenter viewed the scene via a webcam and sent the appropriate commands to the bracelet in order to guide the participant’s hand to the object (Figure 3). During the auditory condition, the experimenter again viewed the scene through the webcam but communicated with the participants using verbal commands. The experimenter started and stopped each trial manually with a button press and afterward indicated in the data whether the participant successfully grasped the target object on that trial or whether the trial was unsuccessful due to mistakes by the experimenter or the participant. While all blocks for each condition were conducted together, participants were allowed a two-minute break between conditions to prevent fatigue. The order of the conditions was counterbalanced, and all other elements of the experiment procedure were kept identical between the two conditions so that the conditions could be validly compared.

All trials followed a standard sequence. At the beginning of the trial, the participant was asked to place their hand on a starting point at the middle shelf’s center (Figure 1A). The target object for each trial was randomly selected such that the participant would grasp each object a total of 8 times per condition but would not grasp every object on each block, a design chosen to act against anticipation on the part of the participant. The participant was always guided along the horizontal axis (left/right) before the vertical axis (up/down), and they received only one direction command at a time. Commands in the auditory condition were given only when the participant needed to start moving their hand, move it in a new direction, or grasp. In the tactile condition, the motor for the selected direction vibrated continuously until a new command was sent. Once the experimenter confirmed through the webcam feed that the participant’s hand was lined up with the target object, they gave the command to grasp either via a series of short pulses on the top motor of the bracelet or verbally (by saying “grasp”), depending on the condition. Contact with the object concluded a trial, and participants were instructed to return to the start position in preparation for the next trial.

For evaluation, we assessed several dependent variables. The main metric of participants’ performance was the mean time to complete a full grasping trial, i.e., the interval between issuing the first command and their first contact with the target object. We also assessed the count of trials with false responses—either the movement of the hand in an incorrect direction or the initiation of grasping without a command from the experimenter. Additionally, we examined the difference in trial completion times across different blocks to assess the presence of a learning effect.

In order to evaluate our results from the grasping task against subjective feedback, we developed a questionnaire for the participants, which they completed at their leisure following the appointment in the lab. This questionnaire included questions regarding the experimental paradigm and the design of the tactile bracelet. Participants first had to respond to each of the 17 statements by selecting a number from 1 (strongly disagree) to 5 (strongly agree) on a Likert scale. To avoid research bias, the order of the statements was randomized individually. The statements were also presented in a balanced framework, with each element being targeted using both positively and negatively formulated statements separately. Two additional open-ended questions asked the participants to describe their general experience with the grasping task, as well as with the tactile bracelet itself, in a freer format that could capture information not assessed directly in the Likert questions. The participants’ answers provided insights into the experimental procedure and into the bracelet itself for consideration in potential follow-up studies and its continued development.

### 2.4. Pilot Study Procedures

To validate the results obtained in the group of blindfolded, normally sighted participants, we tested two visually impaired participants. This validation of the bracelet followed the same procedures as those of the main study. The only difference was that the documentation and instructions were provided in German instead of English, as neither participant spoke English fluently.

Exploring the viability of an AI control system for the bracelet, which would enable its autonomous use, was the subject of a second qualitative pilot study. We investigated various mock users utilizing the bracelet in a paradigm similar to the main experiment. However, here, the bracelet and camera system were controlled via the AI system instead of the experimenter. The mock users provided verbal feedback on the experience of grasping the target objects on the experiment shelf using the bracelet in the fully closed-loop mode under the AI system’s control.

### 2.5. Statistical Analysis

To compare performances in both conditions, we performed hypothesis testing using Welch’s *t*-test to compare group means. The test was applied to determine the main effect of trial time differences between the tactile and auditory conditions, as well as the learning effects between blocks in each condition. Using the same test, we also analyzed whether the order of conditions in which the participants executed the grasping task had a significant effect on the task performance in terms of trial times for each condition. Furthermore, we fitted a linear, mixed-effects model to investigate the influence of the fixed effects of conditions and block numbers, as well as the crossed random effects of the condition order and the experimenter on trial times. Furthermore, using Pearson’s product–moment correlation, we investigated the influence of localization performance on grasping task performance. Finally, to analyze the questionnaire data, we investigated summary statistics for the Likert items, evaluated the qualitative responses in open-ended questions, and ultimately performed principal component analysis (PCA) to infer latent groupings and interpret the group’s summary statistics following [33].

## 3. Results

### 3.1. Localization Task

**Figure 4:**
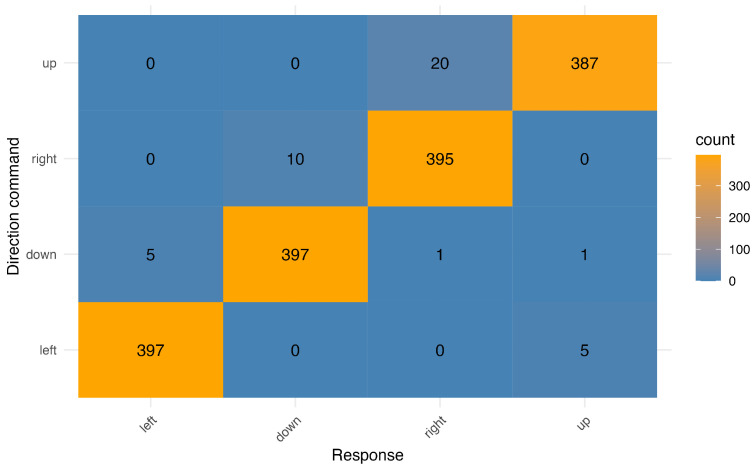
Confusion matrix of vibration directions.

The localization task was conducted to familiarize participants with the bracelet and check whether participants were able to interpret the direction commands well enough to follow them in the grasping task. Twenty-six of the thirty participants (M = 98.07%; SD = 2.78%) achieved the required accuracy of 90% on the first attempt at the task, with the remaining four achieving this by the end of the second attempt (M = 96.34%; SD = 1.99%). None of the participants required a third attempt, and all ultimately passed the accuracy threshold. During the localization task, the bracelet had to be adjusted several times with each participant to fit their unique wrist circumference and bone structure, as vibration motors directly on the radius or ulna bones spread the vibration signal over a wider area and decreased the interpretability of the individual vibration commands. Figure 4 shows how often participants gave a wrong-direction response when a vibration direction was cued, excluding direction commands for which the participants gave no response at all. Across all trials, participants confused up–right directions 20 times, right–down 10 times, left–down five times, up–left five times, and up–down one time. As intended, addressing these confusions in the localization task appeared to assist participants’ performance with the bracelet in the grasping task. A Pearson’s correlation test revealed a significant correlation between accuracy in the localization task and trial time in the grasping task, such that a higher mean localization accuracy was related to lower mean trial times; r(28) = −0.4; *p* = 0.029. These results indicate that the localization task allowed participants to familiarize themselves with the bracelet efficiently and prepared them well for the subsequent grasping task.

### 3.2. Grasping Task

**Figure 5:**
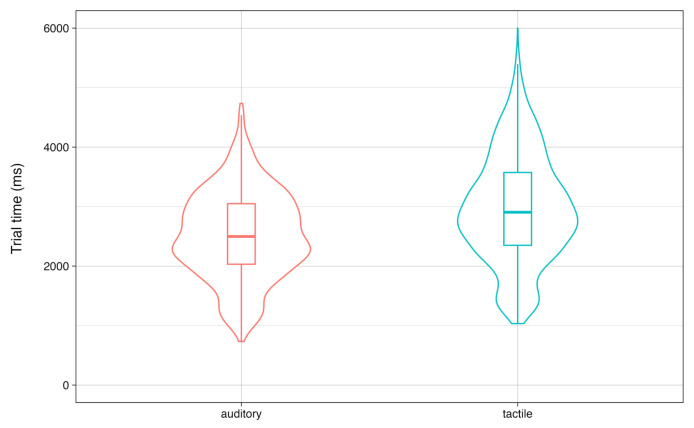
Distribution of trial times on successful trials in the auditory and tactile conditions. The horizontal bar represents the distribution mean.

**Figure 6:**
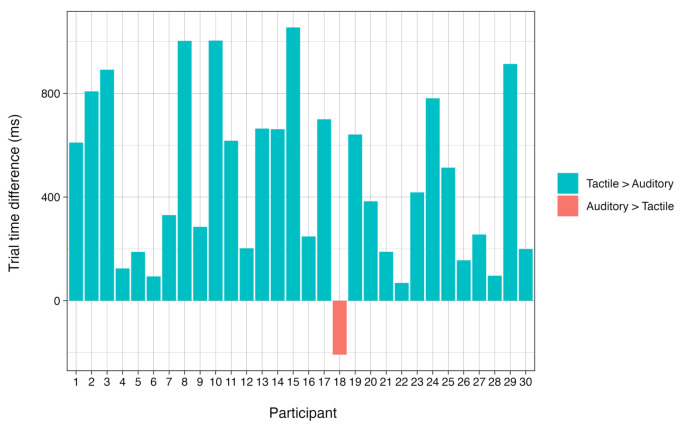
Mean trial times per participant.

To investigate whether the tactile bracelet is a viable alternative to guiding grasping with auditory commands, we assessed how both modalities affected participants’ performance by comparing mean trial times between the auditory and tactile conditions. The distribution of the trial times of the auditory and the tactile conditions overlapped extensively, but the mean trial times in the auditory condition (M = 2538 ms; SD = 757 ms) were significantly shorter compared to the tactile condition (M = 3021 ms; SD = 999 ms), t (3439.2) = −16.5 and *p* < 0.001, with an average difference of 483ms (Figure 5). All except one participant had higher mean trial times in the tactile condition (Figure 6). Although the difference in trial times between the conditions was significant, in view of the total time needed for the grasping movement, the tactile condition faired reasonably well compared to the auditory condition. Further, Participant 18 (the only participant for whom the tactile condition was faster than the auditory condition) reported that they experienced difficulty translating the auditory commands to the correct movement, which was not a factor when following the tactile commands. This indicates that tactile commands may be more effective than auditory commands in cases of common left/right confusion or other verbal command interpretation challenges. Thus, though participants were, on average, somewhat faster in the auditory condition, their performance in the tactile condition qualified it as a viable technique to guide grasping movements.

**Figure 7:**
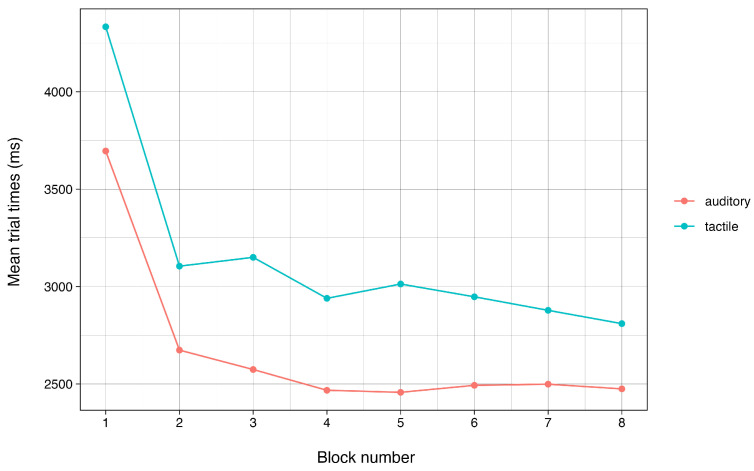
Mean trial times per block grouped by condition.

To further analyze adaptation to the new device, we tested for differences in performance over time. The pattern of performance over time was analogous for both conditions (Figure 7), with the first training block being the slowest in both the auditory (M = 3741 ms; SD = 1817 ms) and tactile (M = 4406 ms; SD = 1944 ms) conditions. The mean trial time declined significantly in the second block of both the auditory (M = 2687 ms; SD = 798 ms), t(371.94) = 8.84 and *p* < 0.001, and tactile (M = 3169 ms; SD = 1126 ms), t (376.33) = 9.21, and *p* < 0.001, conditions. Over the course of the experiment, participants showed steady improvements in both conditions, with mean trial times dropping to 2477 ms (SD = 723 ms) in the auditory condition and 2890 ms (SD = 1000 ms) in the tactile condition for the final block. Thus, participants generally improved in an analogous pattern in both conditions, indicating that participants were able to learn the tactile bracelet and improve with it over time, just as they did with auditory commands.

**Figure 8:**
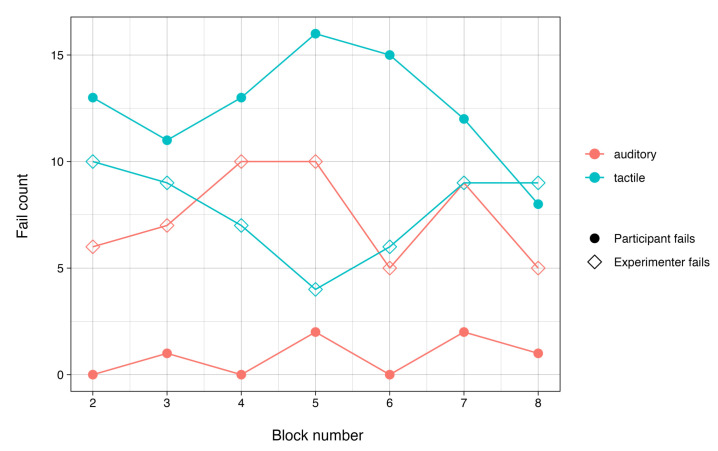
Count of failed trials per block summarized across participants by condition and type of failure.

We further analyzed participants’ ability to learn tactile commands over the course of the study by examining failure counts over time. Figure 8 plots the failure counts over blocks, excluding the practice block for each condition and indicating the number of failures by the experimenter. As expected, the combined count of trials with false responses was significantly lower in the more familiar auditory condition; t (7.38) = −11.13 and *p* < 0.001. It remained comparatively low across all blocks and reached a maximum value of two in blocks five and seven. In the tactile condition, we instead observed a more fluctuating failure count, with the highest number of failures in block five. Failure counts in the tactile condition increased after the third block before steadily declining after the fifth block. These results suggest that, while participants’ accuracy fluctuated over time in the tactile condition compared to the auditory condition, they ultimately improved their performance with the bracelet by the final block.

**Figure 9:**
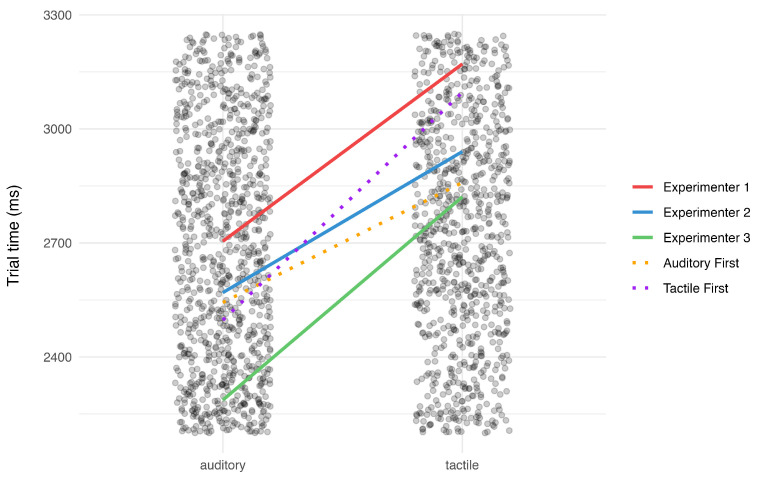
Random effects of condition order and experimenter on trial times. Individual points are a single trial time for a single participant.

As three different experimenters ran the experiment with participants alternately starting in the auditory or tactile condition, we also evaluated the influence of these crossed random effects on the mean trial times. Figure 9 summarizes the findings of the linear mixed-effects model that we ran to test these effects, with the fixed effects of the condition and block number (for the modality and learning effect, respectively) as categorical predictors of the mean trial time. We further included random intercepts and slopes for the specific experimenter and the order of conditions. The effects were compared to the baseline intercept of the mean trial time in the second block of the auditory condition (2667 ms). While the results for the fixed effects of the block number and the condition on the mean trial times largely match the results described in the sections above, the random effects suggest that around 33 ms of variance in the auditory condition are explained by the intercept of an alternating order of conditions, while the slope coefficient accounts for a further 203 ms of variance in the tactile condition. Also, the distribution of work among three experimenters explains around 215 ms of variance in the auditory condition and an additional 92 ms of variance in the tactile condition, indicating that the performance between the experimenters themselves might have influenced the performance of the participants. These results indicate that elements of the study design influenced the performance on a given trial, though the fixed effects of the condition and block number on the mean trial times support the previous findings.

### 3.3. Questionnaire

**Figure 10:**
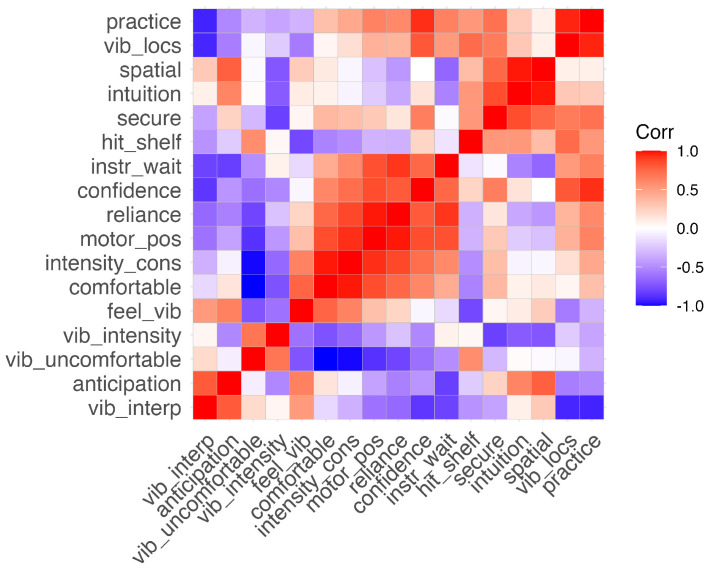
Reordered correlation matrix highlighting the principal component groupings (from left to right: PC1, PC2, and PC3). Abbreviations are noted in Table 1.

**Figure 11:**
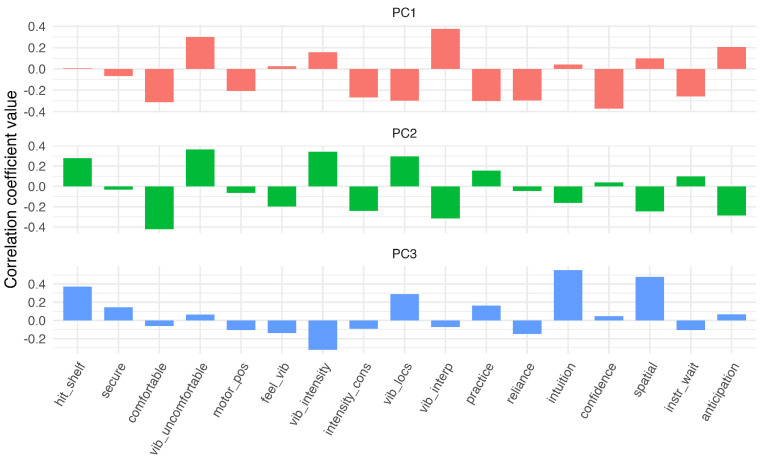
Component loadings of the first three principal components. Abbreviations are noted in Table 1.

To investigate which study elements the Likert questions ultimately generated feedback for and evaluate which factors most influenced participants’ experiences in the study, we performed a PCA to infer latent groupings, following the methodology of [33]. Using a PCA to develop data-driven categories suggested three PCs with a combined 70% explained variance. The groupings ultimately formed through the PCA can be interpreted as feelings with respect to the vibration commands themselves (PC1, 40% explained variance), with respect to the bracelet overall (PC2, 17% explained variance), and with respect to the experiment itself (PC3, 13% explained variance), summarized together in Table 1. The questions split well into the three component groups, with clear differences between them (Figure 10). Figure 11 plots the component loadings for all Likert questions for each PC. This outcome indicates that participants’ experience with the study was largely influenced by how usable and comfortable they found the tactile bracelet to be, with the design of the study and their sense of confidence during the experiment also playing a role.

To compare our quantitative findings with the qualitative evaluations of the participants, we analyzed the questionnaire results with respect to the PCs identified in the PCA, beginning with participants’ feelings regarding the vibration signals themselves. In these analyses, we linked some of the answers to the open-ended questions to some of the corresponding Likert questions. Here, we present the mean and median Likert scores. Participants indicated that the vibration from the bracelet had a consistent intensity, was easy to interpret, and was comfortable on their hand (”The intensity of vibration varied strongly”, M = 1.46 and SD = 0.88; ”It was difficult for me to interpret the vibration cues from the tactile bracelet”, M = 2.36 and SD = 1.03; and ”The vibration from the tactile bracelet felt uncomfortable on my hand”, M = 1.68 and SD = 0.9). These reports indicate that participants generally found the vibrations of the bracelet comfortable and useful in guiding them to the target objects.

We then evaluated the questionnaire responses with respect to participants’ feelings regarding the bracelet overall. Participants reported that the bracelet itself was comfortable with appropriately placed motors whose signals could be felt easily (”The tactile bracelet is comfortable to wear”, M = 3.82 and SD = 1.19; ”The vibration motors were positioned correctly on my arm”, M = 4.18 and SD = 0.98; and ”I could feel the vibration cues on my wrist from the tactile bracelet”, M = 4.75 and SD = 0.52). Further, they indicated that they actually used the bracelet’s signals to guide their hand to the target objects and felt confident in doing so (”I relied on the vibration signals from the tactile bracelet to grasp an object”, M = 4.5 and SD = 0.75; ”I felt confident using the tactile bracelet to locate and grasp an object”, M = 4.11 and SD = 0.99). Overall, therefore, participants reported a positive experience with the bracelet, although they suggested various improvements in the open-ended items of the questionnaire, including a depth-dependent vibration signal, smaller vibration motors, and unique vibration signals for each command. Ultimately, these results indicate that, while the bracelet in its current state is not perfect, it provided a valid platform in this experiment for exploring tactile commands in general, and it warrants further development going forward.

Finally, we evaluated the remaining questionnaire responses related to participants’ feelings with respect to the experiment itself. Participants indicated that the localization task was effective in familiarizing them with the bracelet heading into the main grasping task (”The practice trials were sufficient to get comfortable with using the tactile feedback”, M = 4.39 and SD = 1.03) and that they subsequently felt secure during the grasping task itself without being concerned that they would harm themselves by impacting the shelf or the objects they were interacting with (”I felt secure during the task”, M = 4.43 and SD = 1.03; ”I was in fear of hitting the shelf or objects during the task”, M = 1.68 and SD = 0.98). Participants did report that they developed a mental map of the spatial layout of the shelf (”I was able to develop a spatial understanding of the shelf”, M = 4.64 and SD = 0.62), though they were ambiguous as to whether they then relied on any intuition that this had allowed them to develop when actually grasping the objects (”Mainly my intuition guided my hand movement during the task”, M = 2.64 and SD = 1.1). These reports indicated that participants generally understood the task and were comfortable completing it with the materials and setup used.

**Figure 12:**
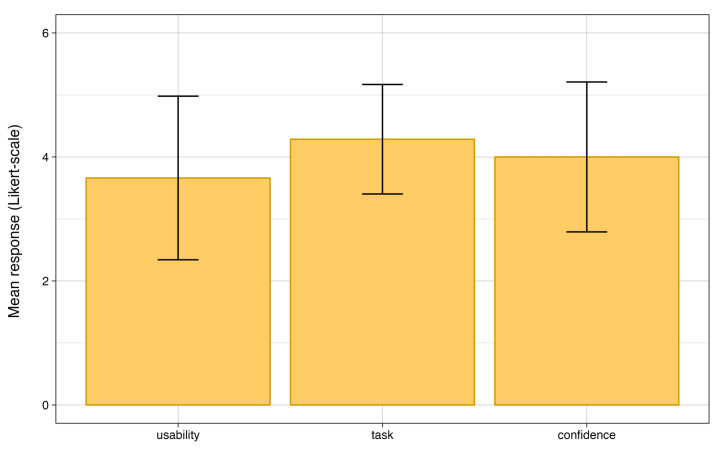
Each construct’s mean participant evaluation.

To evaluate the participants’ overall experience during the study, we investigated the summary statistics of the component groupings above. Figure 12 depicts the participants’ subjective ratings in mean Likert values for each grouping. Participants rated the usability of the bracelet the lowest with a mean of 3.66 (SD = 1.32), potentially reflecting some participants’ difficulties distinguishing the motors. Task design was rated the highest, with an average score of 4.29 (SD = 0.88), indicating a generally good experimental design. Confidence with the bracelet was rated 4.0 on average (SD = 1.21), suggesting that participants were able to learn the bracelet and generally accepted it. Overall, participants rated the whole experience relatively highly (M = 4.04 and SD = 1.14), which serves as confirmation for further developing the bracelet.

### 3.4. Pilot Study with Blind Participants

**Figure 13:**
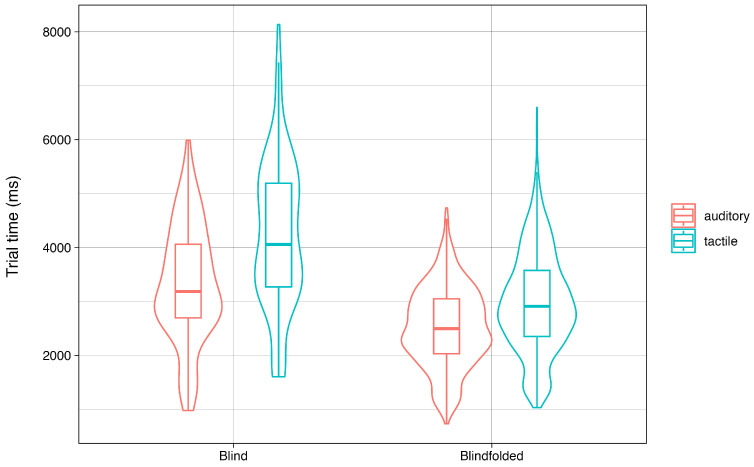
Comparison of successful trial time distributions in the auditory and tactile conditions between blindfolded and blind participants. The violin plot for the blindfolded participants is re-used from Figure 6.

To evaluate how performance with the bracelet might translate to the ultimate target group, we also preliminarily tested the bracelet with two blind participants. The results of both participants mirror the main results from the grasping task and the questionnaire with the blindfolded participants (Figure 13). Quantitatively, the auditory condition was still faster than the tactile condition for them, t (224.69) = −5.74 and *p* < 0.001, but both reported being comfortable with the bracelet and indicated that they were able to understand the commands from it to grasp the target objects. While using only two participants does not provide enough data points to draw substantial conclusions, these results support our previous findings from the study with blindfolded participants, again encouraging further development of the tactile bracelet.

### 3.5. Pilot Study with AI Control System

**Figure 14:**
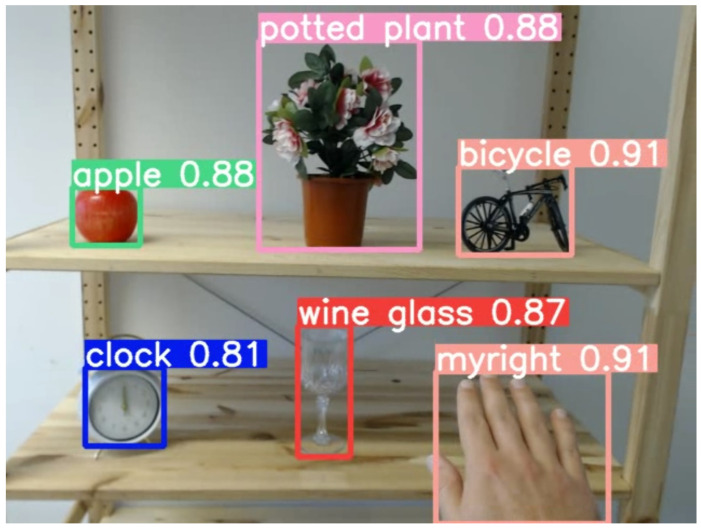
Example of target objects and hand detections.

To evaluate the potential of the bracelet in a closed-loop system without experimenter control, we also tested the bracelet with a group of blindfolded mock users. We implemented a preliminary proof-of-concept AI control system for these tests that consists of two YOLOv5 [34] object detection networks trained on the COCO 2017 [35] and EgoHands 2015 [36] datasets, respectively. These systems reliably identified the target objects and the users’ hands (Figure 14). Using programmed heuristics, the AI control system processes these detections to send the appropriate guiding signals to the users via the tactile bracelet. Specifically, the system generated control signals indicating whether the hand and/or object were in sight and then switched to the spatially guiding signals. The mock users who tested the proof-of-concept AI control system for the bracelet were able to effectively locate and grasp target objects on the shelf while receiving input only from the AI system. However, in some cases, it took repeated trials to interpret the control signals and ensure that the hand and target object were both in the field of view of the camera. Nevertheless, the users provided positive feedback about their experience with this mode. The one suggestion that was repeatedly stated was to separate the control signals and the signals for spatial guidance. Thus, although the AI system used for this pilot study was only a preliminary version, these results demonstrate that the bracelet can also be effectively used in an autonomous mode with no third-party input.

## 4. Discussion

In the localization task, all the participants were able to reach the inclusion criterion, with all of them recording near-ceiling-level performance, indicating that, while novel, the interpretation of tactile direction commands via the bracelet could be learned. Interestingly, the up–right direction was the most frequently confused among participants. As the majority of them were right-handed, this result is likely related to the anatomy of the wrist, in which the radius bone is larger than the ulna [37]. For right-handed users, the bracelet signals indicating movement to the right or up may have, therefore, been more difficult due to the right and up motors both consequently being placed above the same bone, potentially causing localization issues due to bone conduction. The potential implementation of additional motors in the bracelet, smaller motors, more intense vibrations, or more flexibility in the bracelet to adapt to individual wrist structures and sizes are possible solutions to this issue. Though the participants in this study were already able to learn and interpret the vibration signals with a high level of efficacy, implementing these changes in future versions of the bracelet may further improve users’ experiences with the bracelet.

The results from the grasping task show that participants were able to learn and follow the commands from the bracelet during an actual grasping action without losing a great amount of performance compared to working with auditory instructions. Although there was a significant difference between mean trial times between the auditory and tactile modalities, this difference was less than 500 ms. As following auditory commands is a more familiar modality with which participants could be expected to have more experience, it is not surprising that the auditory condition was faster, and the small scale of that difference indicates that tactile guidance may be equally effective as users become more used to the bracelet. This is further supported by the evident learning effect exhibited by the participants over the course of the study, which was also comparable to the learning pattern seen in the auditory condition. Importantly, the number of total trials in the experiment may have facilitated strong learning effects on the task level, but this would have occurred in both conditions. The emergence of comparable learning effect patterns between both conditions shows that participants do not require more time to learn the interpretation of vibrotactile signals than they need to learn auditory commands. Similarly, the current laboratory paradigm also facilitates these learning effects, as the setup only includes the distribution of the target objects on a 3 × 3 grid to simplify navigation, which renders the target locations predictable. However, while a less predictable setup would enable the analysis of participants’ performance in more realistic and comparable environments, it would mainly affect the scale of any task-level learning effect, not the comparability of learning patterns between the two conditions, which was the goal of the present study. While a more complex environment will be needed to better examine learning with the bracelet in more real-world scenarios, the findings in the simplified setting already indicate that navigation with the bracelet can be learned as effectively as auditory commands, with the general learning effect being similar for both modalities. Participants’ trial times in the tactile condition and the number of errors they made decreased throughout the whole task. The one participant who performed faster in the tactile condition than the auditory condition also reported that the tactile commands were easier to interpret, indicating that, in some cases, they may even be more useful and understandable than auditory commands. Further validation of the bracelet in follow-up studies with participants able to undergo further training with the bracelet is, therefore, of great interest to determine whether, with greater training, tactile commands might ultimately match the efficacy of auditory commands or, in some cases, even exceed it.

In the follow-up questionnaire, participants indicated that they found the bracelet usable and were confident using it in the study. Many also provided suggestions for improving the design, which are already being discussed for incorporation in subsequent models. These include adding more vibration motors for better direction representation, modifying signal patterns for different commands, or enabling the adjustment of vibration intensities. A multi-modal approach utilizing pneumatic or wire actuators to enable additional forms of tactile perception [38] and the targeting of cells in the skin selective for specific tactile stimulation to provide users with richer information content [39,40,41] are also being considered. These changes would improve the device, which the questionnaire results already support as a viable grasping assistance tool. Of particular importance are the responses of the two visually impaired participants who completed the study. Both reported being able to effectively use the bracelet, indicating that the bracelet is also a tool of potential value to the target population. This positive feedback indicates that the bracelet is capable of effectively providing users with the orientation information needed to confidently grasp an object and that it is worthy of further development and testing.

To further validate the current tactile bracelet design against a bracelet using auditory cues, we assessed the general differences between the auditory and tactile channels, weighing their advantages and shortcomings in light of our findings. The learning effect demonstrated in this study indicates that new bracelet users are able to adapt to the novel input and perform progressively faster. While the performance using the tactile bracelet was slower than with auditory commands, this difference in reaction times was minor and is a small cost for keeping free the auditory channel, which visually impaired individuals rely on heavily to compensate for their lost visual acuity [42,43,44]. This loss in speed also does not counteract other benefits of the tactile modality, which include the potential for simultaneous use with pre-existing (often auditory-based) aids, such as OrCam [45] or EyeMusic [20], and a reduction in the risk of interfering noise in the environment. Ongoing improvements to the bracelet, such as the use of frequency ranges below 200 Hz to remain in the perceivable range even for users with impaired vibrotactile sensitivity due to age [46] or other factors, may further increase the advantage of tactile stimulation. Once fully developed, the tactile system demonstrated here will leave the critical auditory channel free and offer the user a learnable aid that complements and expands, rather than interferes with, their current, often auditory-based tools and compensation strategies.

In this study, we aimed to establish whether a tactile bracelet might be a viable solution for hand guidance during this critical action. Though auditory signals are common in devices aiming to provide blind and visually impaired individuals with information about their surroundings [47,48], tactile signals are used less frequently. However, such signals have an advantage over the standard auditory commands in that they do not occupy a sensory channel that the visually impaired already heavily rely on to account for their lack of visual input. We showed that participants were able to successfully take direction commands from the bracelet in order to orient their hand towards a target object and successfully grasp it. We, therefore, indicate tactile guidance as a promising avenue for providing the visually impaired with the guidance they need to independently locate and grasp objects that they need to interact with in daily life.

Although the results of this study are promising, it is important to acknowledge the limitations of both the study and the current prototype of the bracelet system, which point to improvements in future work. A key limitation of the current study is that it was conducted on blindfolded participants, while the target users are the blind or visually impaired. There are, of course, fundamental differences between these two populations, with data from previous studies suggesting that their behavior and its underlying mechanisms might differ significantly [49,50]. A particular difference lies in the processing and storing of spatial information, with multiple studies showing that visually impaired people map their surroundings using different strategies than normally sighted people (e.g., [51,52]). Therefore, while the basic principles of the bracelet required initial validation before it was further developed and tested with the target population to avoid the unnecessary use of an especially valuable and limited participant pool, broader-scale testing with visually impaired participants will unquestionably be a vital next step. As the auditory and tactile modalities are preferentially processed by the blind [53,54], their performance with a device conveying information on these channels would not be expected to be lower than that of normally sighted testers, allowing normally sighted participants to provide valid insight into the plausibility of the broader fundamentals of a new design, but this does not mean that testing with normally sighted individuals is valid beyond an evaluation of the basic principles of a new system. Therefore, once we validated the tactile modality in general with the normally sighted participants in the main study presented here, we immediately proceeded with the small target population pilot study to begin the process of moving beyond the validation of fundamental elements of the bracelet’s function and towards the optimization of the device for the population it is intended to help. It is important to note that, for both the blindfolded and blind participants, training itself constitutes a critical role in performance (e.g., [55]) and using the device effectively, and consequently, the effect of the training duration on performance needs to be tested in subsequent experiments. Nonetheless, our preliminary tests with the visually impaired participants in that pilot study indicate the tactile bracelet to also be a viable tool worthy of further development with the target population, though a key step going forward will still, undoubtedly, be a larger-scale test of the bracelet with this population.

A second critical shortcoming of the current prototype concerns independent use. While having the bracelet be experimenter-driven was sufficient for this study’s basic test of the efficacy of tactile guidance, it is not a paradigm that can be scaled up to a useful independent grasping assistance solution. The proof-of-concept AI system tested in the second pilot study indicates that the bracelet can successfully be transitioned to operation within a fully closed-loop paradigm, although further development of the AI control system to better handle more complex scenarios than the shelf setup, such as situations where target objects are partially occluded or placed at varying depths, is needed. The development and testing of a closed-loop system is still in progress, but it is already showing the potential to effectively replace the experimenter and move the bracelet further towards autonomous use.

A final limitation of the bracelet affecting its use in both experimenter-controlled and AI-controlled paradigms is that it only provides guidance on two axes of movement, horizontal and vertical, and it neglects depth. Spatial information about the distance of the object was not of great importance in the laboratory environment of the current study, in which all target objects occurred at the same depth, but it will be crucial for the usability of the bracelet in more complex, real-life environments. The development of functionality that provides additional information about the hand’s distance from the target object, perhaps via a varying vibration intensity or the usage of the bracelet in its current form in parallel with sensory substitution devices (such as that presented in [31]), are key avenues to explore en route to a fully functional grasping support system.

Despite the bracelet’s current limitations, our results validate the tactile channel as an effective modality in guiding movements during grasping and the tactile bracelet itself as an effective device for doing so. Once the current system is enhanced through the integration of the bracelet with the in-progress AI software, it will become an even more realistic option for aiding the grasping processes of the blind and visually impaired. The continued development of the system in light of all the feedback and findings from this study will be ongoing, but similar to what was seen with the naviBelt [28], the bracelet, in its final form, may result in an improvement in perception of the surrounding space by aiding cross-sensory calibration [56], allowing users to gain further independence in their daily lives.

## Figures and Tables

**Table 1:** All questions and their assignments to principal component groupings after PCA.

Principal Component Grouping	Questions
PC1—Vibrations	The intensity of vibration varied strongly. (vib_intensity) The vibration from the tactile bracelet felt uncomfortable on my hand. (vib_uncomfortable) I was anticipating the grasping motion. (anticipation) It was difficult for me to interpret the vibration cues from the tactile bracelet. (vib_interp)
PC2—Bracelet Overall	I could feel the vibration cues on my wrist from the tactile bracelet. (feel_vib) The tactile bracelet is comfortable to wear. (comfortable) I found the vibration intensity to be consistent. (intensity_cons) The vibration motors were positioned correctly on my arm. (motor_pos) I relied on the vibration signals from the tactile bracelet to grasp an object. (reliance) I felt confident using the tactile bracelet to locate and grasp an object. (confidence) I waited for instructions before reaching to grasp an object. (instr_wait)
PC3—Experiment	I was in fear of hitting the shelf or objects during the task. (hit_shelf) I felt secure during the task. (secure) Mainly my intuition guided my hand movement during the task. (intuition) I was able to develop a spatial understanding of the shelf. (spatial) I could identify the vibration locations without much effort. (vib_locs) The practice trials were sufficient to get comfortable with using the tactile feedback. (practice)

## Data Availability

The data presented in this study are openly available at https://osf.io/vu6rb/ (published on 19 January 2024).
